# Obsessive-Compulsive Symptoms During Pregnancy and the Postpartum Period: A Cross-Sectional Comparison with Non-Perinatal Female Controls During the COVID-19 Pandemic

**DOI:** 10.3390/jcm15218665

**Published:** 2026-11-06

**Authors:** Jana Christina Müller, Laura Michalke, Amir-Hosseyn Yassari, Franziska Miegel, Heiko Albrecht, Lena Jelinek

**Affiliations:** Department of Psychiatry and Psychotherapy, University Medical Center Hamburg-Eppendorf, Martinistraße 52, 20246 Hamburg, Germany

**Keywords:** obsessive–compulsive symptoms, pregnancy, postpartum, COVID-19, depression, anxiety

## Abstract

**Background:** Pregnancy and the postpartum period have been associated with increased vulnerability to obsessive–compulsive symptoms (OCSs), while the COVID-19 pandemic introduced additional psychological stressors. This study compared self-reported OCSs, depressive symptoms, and anxiety symptoms among pregnant, postpartum, and non-perinatal female controls during the COVID-19 pandemic. **Methods:** In this cross-sectional online study, 203 women were assessed between June 27 and August 9, 2021, including 66 pregnant women, 53 women who had given birth within the preceding 12 months, and 84 non-perinatal female controls. Symptoms were assessed using the Obsessive–Compulsive Inventory—Revised (OCI-R), Patient Health Questionnaire-9 (PHQ-9), and Generalized Anxiety Disorder-7 (GAD-7). The OCI-R recall period was modified to seven days, and its thresholds were used for exploratory categorization. Group differences were examined using analyses of variance and chi-square tests, supplemented by exploratory adjusted analyses. **Results:** Overall, 35.5% of participants scored at or above the exploratory OCI-R threshold of 18, while 30.2% and 21.3% scored at or above established screening thresholds for depressive and anxiety symptoms, respectively. The proportions did not differ significantly between groups for OCI-R ≥ 18 (*p* = 0.804), PHQ-9 ≥ 10 (*p* = 0.892), or GAD-7 ≥ 10 (*p* = 0.506). Continuous symptom scores also showed no statistically significant group differences. The proportion scoring at or above the exploratory OCI-R threshold of 28 was descriptively higher among postpartum women (22.6%) than among pregnant (10.6%) or non-perinatal female controls (9.5%), but the overall group comparison was not statistically significant (*p* = 0.065). **Conclusions:** No statistically significant differences in general self-reported OCSs, depressive symptoms, or anxiety symptoms were detected between pregnant, postpartum, and non-perinatal women. These findings do not establish equivalent symptom burden or exclude perinatal vulnerability to specific obsessive–compulsive symptoms. Larger longitudinal studies using validated perinatal-specific assessments are needed.

## 1. Introduction

The perinatal period has been associated with an increased vulnerability to the onset or exacerbation of obsessive–compulsive disorder (OCD) and obsessive–compulsive symptoms (OCSs) [1,2,3]. Several characteristics of the transition to motherhood may contribute to this vulnerability, including new responsibilities, increased uncertainty, and changes in everyday life [4]. Prospective findings showed that obsessive beliefs during pregnancy, particularly those related to responsibility and threat, predicted subsequent postpartum OCSs [5].

A recent meta-analysis estimated a pooled OCD prevalence of 9.1% during pregnancy and 6.2% during the postpartum period [6]. In a prospective study following women from pregnancy to six months postpartum, Fairbrother et al. (2021) found a weighted OCD period prevalence of 7.8% during pregnancy and 16.9% during the postpartum period [2]. However, reported rates of OCSs vary considerably across studies. This may partly be related to differences in samples and assessment methods [3]. The COVID-19 pandemic was associated with considerable psychological burden, including obsessive–compulsive, depressive, and anxiety symptoms [7,8]. Given the previously described vulnerability during the perinatal period, this raised the question of whether pregnant and postpartum women experienced greater psychological symptom burden than non-perinatal female controls under comparable pandemic conditions. Several studies reported elevated symptoms in perinatal populations. Mahaffey et al. (2022) found that 7.12% of pregnant participants had clinically relevant OCS scores and that pandemic-related pregnancy stress was associated with higher OCSs [9]. Yassa et al. (2020) reported higher obsessive–compulsive symptom scores among pregnant than non-pregnant women, whereas state anxiety scores were higher in the non-pregnant group [10]. Thompson and Bardone-Cone (2021) found higher levels of specific OCD-type symptoms related to contamination and responsibility for harm among postpartum women compared with controls, despite no statistically significant differences in overall OCD-type, depressive, or general anxiety symptom scores after correction for multiple comparisons [11]. Elevated levels of anxiety and depressive symptoms were also reported in perinatal populations during the pandemic [12]. However, the broader evidence has been heterogeneous: A systematic review identified mixed findings in comparisons of psychological symptoms between pregnant and non-pregnant women [13]. Penengo et al. (2022) reported no significant differences in anxiety, depression, or OCD screening outcomes across the pandemic periods examined among pregnant women [14], while Tauqeer et al. (2023) reported similar proportions of elevated anxiety and depressive symptoms among pregnant and postpartum women [15]. Taken together, these findings suggest that observed differences may depend on the symptom domain examined, the populations compared, and the timing and methods of assessment. Importantly, elevated symptom levels within perinatal samples, similarities between pregnant and postpartum women, or comparisons across pandemic periods do not necessarily establish whether perinatal women experience greater symptom burden than non-perinatal female controls assessed concurrently.

A concurrent three-group comparison may therefore help clarify whether differences in self-reported obsessive–compulsive, depressive, and anxiety symptom scores are detectable across these reproductive stages. In the present cross-sectional study, we compared these symptom scores among pregnant, postpartum, and non-perinatal female controls during the COVID-19 pandemic. Based on previous evidence suggesting increased perinatal vulnerability, we hypothesized higher levels of obsessive–compulsive, depressive, and anxiety symptoms in pregnant and postpartum women than in non-perinatal female controls.

## 2. Methods

### 2.1. Recruitment and Procedure

Women aged 18–50 years were recruited between 27 June 2021, and 9 August 2021, through social media platforms (e.g., Facebook and Twitter) and gynecology practices (e.g., via leaflets). The recruitment channel through which individual participants entered the study was not recorded. Recruitment materials provided a short description of the study aims and a link to the online survey, which was conducted via Qualtrics XM^®^. At the beginning of the survey, participants provided electronic informed consent. Subsequently, demographic information (e.g., age and education), pregnancy-related information, and psychiatric history were assessed. Participants then completed the Obsessive–Compulsive Inventory—Revised (OCI-R) [16], the Patient Health Questionnaire-9 (PHQ-9) and the Generalized Anxiety Disorder Screener (GAD-7) [17,18,19,20].

Inclusion criteria were (1) age between 18 and 50 years, (2) female gender, and (3) the completion of all the OCI-R items. “Cookies” prevented multiple participation from the same computer. Participants received a link to download a PDF manual on relaxation techniques as compensation for their participation.

### 2.2. Participants

A total of 263 participants accessed the survey. Of these, 30 did not provide informed consent or agree to the data security settings. One participant did not meet the gender inclusion criterion, and 28 participants did not complete the OCI-R. In addition, responses were screened for stereotypical response patterns in their psychopathology measures (i.e., entering the same value throughout the psychopathology assessment), resulting in the exclusion of one participant. Participants were allocated to groups based on whether they were currently pregnant or had given birth within the previous 12 months. The postpartum group comprised women who had given birth within the preceding 12 months. This time frame was selected to capture mental health symptoms across the first year after childbirth, consistent with the scope of established guidance on antenatal and postnatal mental health [21].

The final sample comprised 203 participants: 66 pregnant women, 53 postpartum women, and 84 non-perinatal female controls.

### 2.3. Assessment

#### 2.3.1. Obsessive–Compulsive Inventory—Revised (Oci-R)

The OCI-R consists of 18 items indexing the severity of OCSs across six subscales: washing, obsessing, checking, neutralizing, hoarding, and ordering [16]. Items are rated on a 5-point Likert-scale ranging from 0 = not at all to 4 = very much, resulting in a total score ranging from 0 to 72. For the present study, the OCI-R recall period was shortened to the preceding seven days to assess recent OCSs within a defined and relatively brief time frame. This modification was intended to capture current symptom burden during the period of data collection. However, the modification represents a departure from the validated administration of the instrument and may affect the comparability of scores with those obtained using the original recall period. Score ranges of 0–15, 16–27, and 28–72 have been described as reflecting mild, moderate, and severe obsessive–compulsive symptom levels, respectively, in previous research [22].

The German version of the OCI-R has demonstrated good reliability and validity in clinical samples, including patients with OCD, anxiety disorders, and depressive disorders [23,24]. However, psychometric validation specifically in German-speaking pregnant and postpartum populations has not been established. In the present sample, the OCI-R total score showed good internal consistency (Cronbach’s α = 0.855). The conventional OCI-R thresholds of ≥18 and ≥28 were retained for descriptive and exploratory purposes. Because these thresholds were established using the original instrument, their validity for the modified seven-day recall period has not been established. Accordingly, they were treated as exploratory score categories rather than validated indicators of clinically significant OCSs or diagnostic severity.

#### 2.3.2. Patient Health Questionnaire-9 (Phq-9)

The German version of the PHQ-9 was used to assess the severity of depressive symptoms [19]. The questionnaire has demonstrated good validity and reliability [25,26]. The nine items assess depressive symptoms during the previous two weeks and are rated on a 4-point Likert-scale ranging from 0 = not at all to 3 = nearly every day, resulting in a total score ranging from 0 to 27. Scores of 5, 10, 15, and 20 points represent thresholds for mild, moderate, moderately severe, and severe depressive symptoms, respectively [25]. A cut-off score of ≥10 was used to indicate clinically relevant depressive symptoms [25].

#### 2.3.3. Generalized Anxiety Disorder Screener (Gad-7)

The German version of the GAD-7 was used to assess anxiety symptoms [17,20]. The seven items assess symptoms during the previous two weeks and are rated on a 4-point Likert scale ranging from 0 = not at all to 3 = nearly every day, resulting in a total score ranging from 0 to 21. Scores of 0–4, 5–9, 10–14, and 15–21 indicate minimal, mild, moderate, and severe anxiety symptoms, respectively. A cut-off score of ≥10 was used to indicate clinically relevant anxiety symptoms [17].

### 2.4. Strategy of Data Analysis

Group differences in sociodemographic characteristics and psychiatric history were examined using one-way ANOVAs for continuous variables (e.g., age), Kruskal–Wallis tests for ordinal variables (e.g., income), and χ^2^ tests for categorical variables (e.g., lifetime history of mental illness). Participants included in the final sample and participants who did not complete the survey were compared regarding age and number of children using independent-samples *t*-tests.

Group differences in the proportions of participants scoring at or above the exploratory OCI-R thresholds of ≥18 and ≥28, as well as the established screening thresholds for depressive symptoms (PHQ-9 ≥ 10) and anxiety symptoms (GAD-7 ≥ 10), were examined using χ^2^ tests.

Cramer’s V was calculated as an effect size. For the exploratory OCI-R thresholds of ≥18 and ≥28, 95% confidence intervals for the observed proportions were calculated using the Wilson score method. Unadjusted 95% confidence intervals for pairwise differences in proportions were calculated using the Newcombe method for independent proportions.

Separate one-way ANOVAs with group (pregnant, postpartum, and non-perinatal female controls) as the between-subjects factor were conducted for the OCI-R total score and subscale scores, PHQ-9 total score, and GAD-7 total score. In addition, Bonferroni-corrected *t*-tests were conducted as planned pairwise comparisons between the three groups.

As exploratory analyses, the ANOVAs were repeated for (1) married women or women in a stable relationship, (2) women with a monthly income > €1000, and (3) non-students. To complement these analyses, exploratory analyses of covariance (ANCOVAs) were conducted to examine group differences in symptom scores after adjustment for selected sociodemographic characteristics. The first model adjusted for age and relationship status (stable relationship: yes/no). The second model additionally adjusted for the number of children. Separate ANCOVAs were conducted for the OCI-R total score, PHQ-9 total score, and GAD-7 total score. Effect sizes were calculated as partial η^2^ for the ANOVAs and ANCOVAs (small: ≈0.01, medium: ≈0.06, large: ≈0.14) and Cohen’s d for the pairwise comparisons (small: 0.20, medium: 0.50, large: 0.80). Analyses were based on available data for each outcome measure. All analyses were conducted using IBM SPSS Statistics, version 26.

An a priori sample size calculation was performed during study planning. However, the original calculation and its underlying assumptions are no longer available and therefore cannot be reliably reported. To characterize the sensitivity of the achieved sample, we conducted a supplementary sensitivity analysis using G*Power (Version 3.1.9.6.; Heinrich-Heine-Universität Düsseldorf, Düsseldorf, Germany). With a total sample size of *N* = 203, three groups, α = 0.05, and 80% power, the analysis indicated a minimum detectable effect size of approximately f = 0.22 (partial η^2^ ≈ 0.046) for the omnibus group comparison.

### 2.5. Use of Generative Artificial Intelligence

During manuscript revision, ChatGPT (OpenAI, GPT-6 version; OpenAI, San Francisco, CA, USA) was used to assist with English-language editing, improving the clarity and consistency of scientific reporting, and reviewing the alignment of the Methods, Results, Discussion, and Supplementary Materials. It was also used to support the development of SPSS syntax for verifying selected descriptive statistics and to critically review the presentation and interpretation of statistical findings. All statistical analyses and calculations were performed and verified by the authors using the original study data in SPSS. All AI-assisted suggestions were critically evaluated by the authors, who take full responsibility for the accuracy and final content of the manuscript. Generative AI was not used for study design, participant recruitment, or data collection.

## 3. Results

### 3.1. Demographics and Psychopathology

The final sample is described in Table 1. When comparing the final sample with participants who did not complete the survey, no differences were found in age (*t*(231) = 0.826, *p* = 0.410) or number of children (*t*(228) = 0.111, *p* = 0.911). Gestational age was available for 65 of the 66 pregnant participants. Mean gestational age was 25.05 weeks (SD = 11.57; range: 3–41 weeks), with a median of 30 weeks. Of these participants, 18 (27.7%) were in the first trimester (≤13 weeks), 10 (15.4%) in the second trimester (14–27 weeks), and 37 (56.9%) in the third trimester (≥28 weeks). The non-perinatal female controls included both women without children (*n* = 38, 45.2%) and women with at least one child (*n* = 46, 54.8%). Additionally, 20 women (23.8%) in this group reported a desire to have children. Pregnant women, postpartum women, and non-perinatal female controls did not differ in age, income, or lifetime history of a mental illness, but differences were found in the number of children, marital status, and occupational status (see Table 1).

### 3.2. Symptoms Above Published Cutoff Scores

Using the exploratory OCI-R threshold of ≥18, 35.5% (*n* = 72) of the final sample scored at or above this threshold. In addition, 21.3% (*n* = 43/202) scored at or above the cut-off for clinically relevant anxiety symptoms (GAD-7 ≥ 10), and 30.2% (*n* = 60/199) scored at or above the screening threshold for clinically relevant depressive symptoms (PHQ-9 ≥ 10).

**Figure 1:**
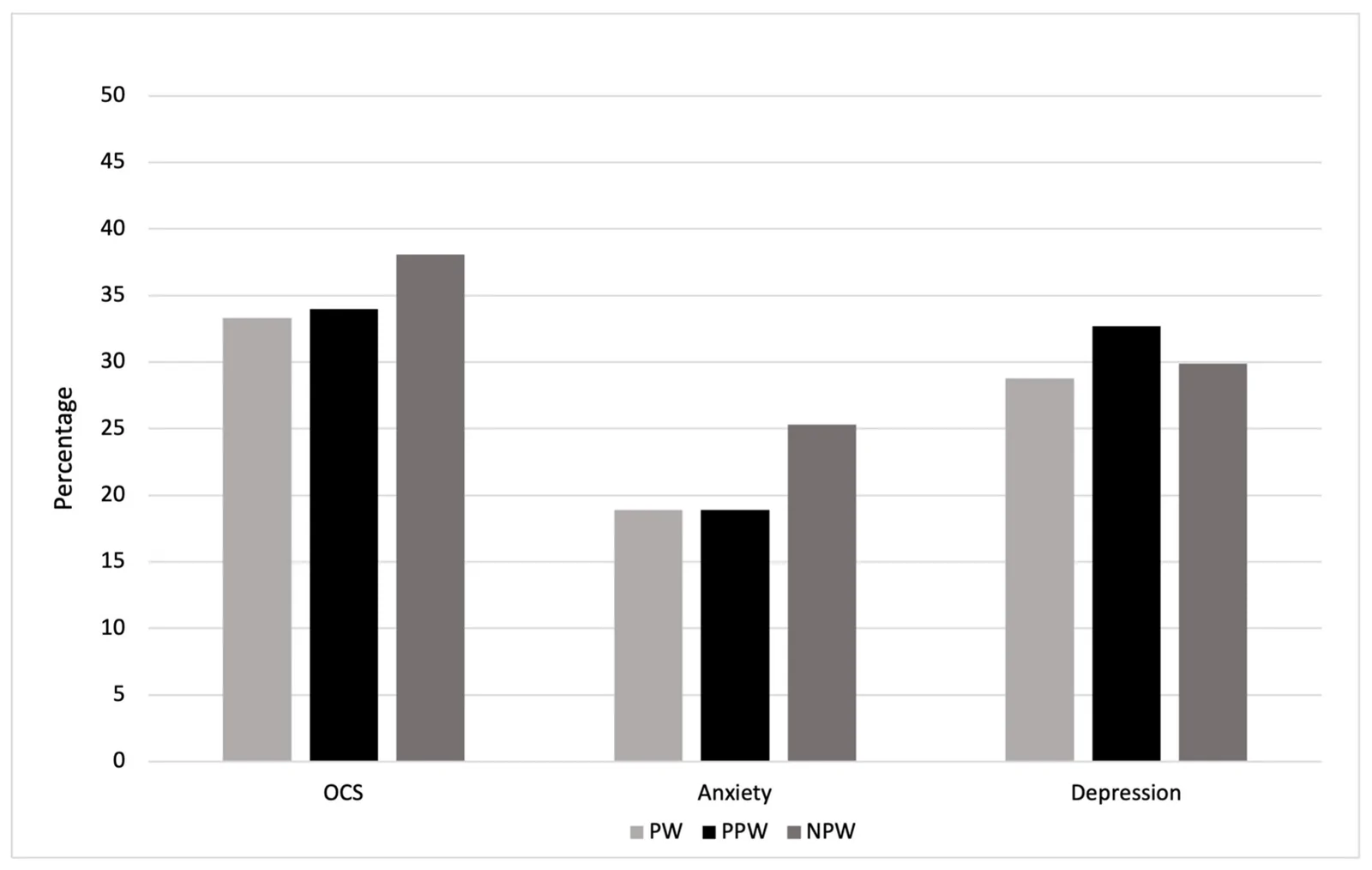
Percentages of pregnant women (PW), postpartum women (PPW), and non-perinatal female controls (NPW) scoring at or above the exploratory OCI-R threshold of 18 or the established screening thresholds for anxiety (GAD-7 ≥ 10) and depressive symptoms (PHQ-9 ≥ 10).

The proportions did not differ significantly between pregnant women, postpartum women, and non-perinatal female controls for the exploratory OCI-R threshold of ≥18 (Cramer’s V = 0.046, *p* = 0.804), the GAD-7 screening threshold of ≥10 (Cramer’s V = 0.082, *p* = 0.506), or the PHQ-9 screening threshold of ≥10 (Cramer’s V = 0.034, *p* = 0.892; see Figure 1).

Using the exploratory OCI-R threshold of ≥28, 27 of 203 participants (13.3%, 95% CI [9.3%, 18.7%]) scored at or above this threshold. The corresponding proportions were 10.6% among pregnant women (7/66; 95% CI [5.2%, 20.3%]), 22.6% among postpartum women (12/53; 95% CI [13.5%, 35.5%]), and 9.5% among non-perinatal female controls (8/84; 95% CI [4.9%, 17.7%]). The overall group comparison was not statistically significant, χ^2^(2) = 5.465, *p* = 0.065, Cramer’s V = 0.164. Pairwise differences in the proportions of participants scoring at or above the exploratory OCI-R thresholds of 18 and 28, together with their unadjusted 95% confidence intervals, are presented in Supplementary Table S2. For the threshold of ≥18, all confidence intervals included zero. For the threshold of ≥28, the postpartum group showed a higher proportion than the non-perinatal female controls (difference = 13.1 percentage points, 95% CI [0.8, 26.8]). However, this unadjusted exploratory finding should be interpreted cautiously in light of the nonsignificant overall group comparison, multiple testing, and the modified OCI-R recall period.

### 3.3. Symptom Severity

Means and standard deviations for the OCI-R total and subscale scores, GAD-7, and PHQ-9 are presented in Table 2. The one-way ANOVAs indicated no significant group differences across these measures, with partial η^2^ ranging from 0.001 to 0.014.

### 3.4. Pairwise Comparisons and Sensitivity Analyses

Bonferroni-adjusted pairwise comparisons of OCI-R total scores revealed no statistically significant differences between pregnant and postpartum women (mean difference = −1.15, t(200) = −0.65, 95% CI [−5.42, 3.12], *p* = 1.000), pregnant women and the non-perinatal female controls (mean difference = −0.88, t(200) = −0.56, 95% CI [−4.69, 2.93], *p* = 1.000), or postpartum women and the non-perinatal female controls (mean difference = 0.27, t(200) = 0.16, 95% CI [−3.79, 4.33], *p* = 1.000). These findings do not establish equivalence between groups.

Sensitivity analyses were conducted in subsamples restricted to (1) women who were married or in a stable relationship, (2) women with a monthly income > €1000, and (3) non-students. Across these subsamples, group comparisons of OCI-R total and subscale scores, GAD-7 scores, and PHQ-9 scores remained statistically nonsignificant (all *p* ≥ 0.251; see Supplementary Table S3).

In complementary exploratory analyses, ANCOVAs adjusting for age and relationship status showed no statistically significant group effects on OCI-R total scores, F(2, 198) = 0.316, *p* = 0.730, partial η^2^ = 0.003; GAD-7 scores, F(2, 197) = 0.531, *p* = 0.589, partial η^2^ = 0.005; or PHQ-9 scores, F(2, 194) = 0.343, *p* = 0.710, partial η^2^ = 0.004. Group effects also remained nonsignificant when the number of children was additionally included in the models (OCI-R: F(2, 197) = 0.521, *p* = 0.595, partial η^2^ = 0.005; GAD-7: F(2, 196) = 0.556, *p* = 0.575, partial η^2^ = 0.006; PHQ-9: F(2, 193) = 0.179, *p* = 0.836, partial η^2^ = 0.002). Detailed results are presented in Supplementary Table S1.

## 4. Discussion

The present study compared OCSs, depressive symptoms, and anxiety symptoms among pregnant, postpartum, and non-perinatal female controls during the COVID-19 pandemic. Contrary to our hypotheses, we did not detect statistically significant group differences in these symptom domains, either in continuous symptom scores or in the proportions scoring at or above the applied thresholds. The exploratory analyses adjusting for selected sociodemographic characteristics yielded a similar pattern. However, these findings should not be interpreted as evidence of equivalent symptom burden across groups, and residual confounding cannot be excluded. Nevertheless, elevated symptom scores were common across the total sample: 35.5% scored at or above the exploratory OCI-R threshold of 18, while 30.2% and 21.3% met the established screening thresholds for depressive and anxiety symptoms, respectively. Because the OCI-R recall period was modified to seven days, the proportion scoring at or above its published threshold cannot be interpreted as a validated estimate of clinically relevant OCSs. A descriptively higher proportion of postpartum women scored at or above the exploratory OCI-R threshold of 28. However, the overall comparison of proportions was not statistically significant (*p* = 0.065), the confidence intervals indicated considerable uncertainty, and the confidence intervals for pairwise differences in proportions were not adjusted for multiple comparisons. This observation should therefore be regarded as hypothesis-generating rather than evidence of a confirmed group difference.

Our findings on OCSs differ from some previous studies indicating an increased vulnerability to OCD and OCSs during pregnancy and the postpartum period [2,3], including studies conducted during the COVID-19 pandemic [10,11]. However, reported rates of OCD and OCSs during pregnancy and the postpartum period vary considerably between studies, which may partly be related to differences in the investigated samples and assessment methods [3]. Thus, our findings do not rule out an increased vulnerability to OCSs during the perinatal period.

Data collection took place in Germany between 27 June and 9 August 2021, during a period of easing COVID-19 restrictions and changing epidemiological conditions. Following a decline in infection rates after the third pandemic wave, the nationwide emergency restrictions expired on 30 June 2021, although other protective measures remained in place, and infection rates increased again over the following weeks [27,28]. However, the present cross-sectional data do not allow us to determine whether pandemic-related circumstances influenced psychological symptom levels or group differences. The pandemic should therefore be considered part of the historical context of data collection rather than an established explanation for the absence of statistically significant group differences.

A further consideration is the distinction between general OCS severity and the specific content and context of symptoms during the perinatal period. Although no statistically significant group differences were detected in the OCI-R total score or its six subscales, the OCI-R does not specifically assess concerns related to pregnancy, childbirth, or infant care [16]. Previous studies have reported perinatal-specific symptom patterns, including aggressive obsessions, concerns about accidental harm to the infant, checking, and reassurance seeking [29]. It is therefore conceivable that such differences in symptom content may not be adequately captured by general OCS measures such as the OCI-R [30]. However, because the present study did not assess the specific content of OCSs, this interpretation remains speculative. Future studies should therefore assess perinatal-specific thoughts and behaviors alongside general OCSs, for example using the Perinatal Obsessive–Compulsive Scale (POCS) [31]. Beyond these implications for future research, the findings also highlight the importance of considering OCSs in clinical assessments of women both during and outside the perinatal period. When such symptoms are reported, clinicians should consider further assessment of symptom severity, associated distress, functional impairment, and potential need for support, rather than relying on questionnaire thresholds alone.

Elevated depressive and anxiety symptom scores were also common in our sample. Previous studies reported considerable psychological burden among pregnant and postpartum women during the COVID-19 pandemic [12]. In the present study, no statistically significant group differences were detected in depressive or anxiety symptom scores. However, these findings should be interpreted cautiously and should not be taken as evidence that meaningful differences between the groups are absent.

### 4.1. Strengths and Limitations

One strength of the present study is the direct comparison of pregnant women, postpartum women, and a non-perinatal female controls within the same sample. Furthermore, OCSs, depressive symptoms, and anxiety symptoms were assessed using established self-report questionnaires. Nevertheless, some methodological limitations must be considered.

First, the study relied on self-report questionnaires without clinician-administered diagnostic interviews. Accordingly, screening results cannot establish psychiatric diagnoses. Furthermore, although lifetime psychiatric history was assessed, detailed information on specific psychiatric diagnoses, previous OCD, current psychiatric treatment, and psychotropic medication use was not available. Consequently, we could not determine whether pre-existing OCD or other active psychiatric disorders contributed to the observed symptom scores. Although the OCI-R has been examined in perinatal samples, its conventional cutoff scores have not been specifically validated for identifying clinically relevant OCSs or OCD in pregnant and postpartum women [9]. An additional measurement limitation concerns the modification of the OCI-R recall period to the preceding seven days. The psychometric properties and conventional thresholds of the OCI-R were established for the original instrument and cannot be assumed to apply unchanged to this modified version. While internal consistency was good in the present sample, this does not establish the validity of the modified instrument or its conventional cutoffs. Accordingly, OCI-R scores should be interpreted as self-reported OCS burden, and the thresholds of 18 and 28 should be regarded as exploratory score categories rather than validated indicators of OCD prevalence or clinical severity. The shorter recall period may also affect the comparability of OCI-R scores with previous studies using the original instrument, although the direction and magnitude of this effect are unknown.

Second, although an a priori sample size calculation was conducted, its original assumptions could not be retrieved. The subgroup sizes were relatively small, particularly for pregnant women (*n* = 66) and postpartum women (*n* = 53), limiting the precision of estimates and the ability to detect potentially meaningful differences. A sensitivity analysis for the three-group omnibus ANOVA (*N* = 203, α = 0.05, power = 0.80) indicated a minimum detectable effect of approximately f = 0.22 (corresponding to partial η^2^ ≈ 0.046). Thus, the study may have been insufficiently sensitive to detect smaller group differences, and the absence of statistically significant findings should not be interpreted as evidence of equivalence between groups. The relatively wide confidence interval for the proportion of postpartum women scoring ≥28 on the OCI-R further illustrates the uncertainty of the descriptive estimates.

Third, the postpartum group included women at different stages within the first year after childbirth, during which psychological symptoms and their determinants may vary. Unfortunately, the time elapsed since delivery was not recorded, preventing us from distinguishing between early and later postpartum stages or examining potential associations with symptom scores. Information on adverse birth outcomes was also unavailable, although such experiences may be associated with psychological symptom burden during the postpartum period. This heterogeneity limits the interpretation of the descriptively higher proportion of postpartum participants scoring at or above the exploratory OCI-R threshold of 28. For most pregnant participants, gestational age was available, but the study was not designed to provide adequately powered comparisons across pregnancy stages. In addition, the non-perinatal female controls were heterogeneous with respect to parental status and desire to have children. Information on active attempts to conceive, infertility, and previous pregnancy loss was not available. Taken together, these unmeasured reproductive experiences may be associated with psychological symptom burden and could not be considered in the group comparisons.

Fourth, participants were recruited through voluntary participation, including social media and gynecology practices. Recruitment channels were not systematically recorded at the individual level, preventing assessment of differences by recruitment source. Selection bias is therefore possible, and the sample cannot be considered representative of pregnant, postpartum, or non-perinatal women in Germany.

Finally, the cross-sectional design precludes conclusions about symptom trajectories or causal relationships. No pre-pandemic measurements were available, and individual exposure to specific pandemic-related circumstances was not systematically assessed. Therefore, we cannot determine whether the observed symptom scores were elevated because of the pandemic or whether pandemic-related circumstances influenced differences between groups. Future studies should use larger, more representative longitudinal samples, document gestational age and time since delivery, and employ clinician-administered and validated perinatal-specific assessments of OCSs.

### 4.2. Conclusions

In this cross-sectional study, we did not detect statistically significant differences in self-reported obsessive–compulsive, depressive, or anxiety symptom scores between pregnant women, postpartum women, and non-perinatal female controls. However, these findings should not be interpreted as evidence of equivalent symptom burden or as excluding increased vulnerability to OCSs during pregnancy and the postpartum period. The relatively small subgroup sizes, heterogeneity within the postpartum group and non-perinatal female controls, and limitations of the modified OCI-R assessment warrant cautious interpretation. Larger longitudinal studies using validated perinatal-specific assessments are needed to clarify potential differences in both overall OCS severity and symptom content across perinatal stages.

## Figures and Tables

**Table 1:** Demographics and Psychopathology: Means (Standard Deviations) or Frequencies (Percentages) for the Total Sample and the Subsamples (Pregnant Women (PW), Postpartum Women (PPW), and Non-Perinatal Female Controls (NPW)).

Variable	Total Sample	PW	PPW	NPW	
	*N* = 203	*n* = 66	*n* = 53	*n* = 84	Statistics
Age (years)	31.75 (5.44)	31.95 (4.47)	31.92 (3.96)	31.48 (6.82)	*F*(2, 200) = 0.18, *p* = 0.840
Income (€)					
<1000	31 (15.27)	5 (7.58)	6 (11.32)	20 (23.81)	*H*(2) = 4.992, *p* = 0.082
1000–2000	39 (19.21)	10 (15.15)	12 (22.64)	17 (20.24)	
2000–3000	30 (14.78)	12 (18.18)	6 (11.32)	12 (14.29)	
3000–4000	36 (17.73)	14 (21.21)	11 (20.76)	11 (13.1)	
>4000	55 (27.09)	23 (34.85)	16 (30.19)	16 (19.05)	
No answer	12 (5.91)	2 (3.03)	2 (3.77)	8 (9.52)	
Occupational status					
None	2 (0.99)	0 (0.00)	0 (0.00)	2 (2.38)	*p* < 0.001, Cramer’s V = 0.493
Part-time	43 (21.18)	16 (24.24)	2 (3.77)	25 (29.76)	
Full-time	35 (17.24)	16 (24.24)	2 (3.77)	17 (20.24)	
Student	25 (12.32)	1 (1.52)	1 (1.89)	23 (27.38)	
Sick leave (>6 weeks)	5 (2.46)	3 (4.55)	0 (0.00)	2 (2.38)	
Parental leave	87 (42.86)	27 (40.91)	48 (90.57)	12 (14.29)	
Housewife	5 (2.46)	2 (3.03)	0 (0.00)	3 (3.57)	
Retired	1 (0.49)	1 (1.52)	0 (0.00)	0 (0.00)	
Family status					
Single	22 (10.84)	6 (9.09)	0 (0.00)	16 (19.05)	*p* = 0.003, Cramer’s V = 0.221
Serious relationship	48 (23.65)	18 (27.27)	9 (16.98)	21 (25.00)	
Divorced	2 (0.99)	0 (0.00)	0 (0.00)	2 (2.38)	
Married	131 (64.53)	42 (63.64)	44 (83.02)	45 (53.57)	
Children					
0	70 (34.48)	32 (48.48)	0 (0.00)	38 (45.24)	*H*(2) = 30.515, *p* < 0.001; PW vs. NPW: *p* = 0.308 PW vs. PPW: *p* < 0.001 NPW vs. PPW: *p* < 0.001
1	79 (38.92)	27 (40.91)	31 (58.49)	21 (25.00)	
2	42 (20.69)	4 (6.06)	19 (35.85)	19 (22.62)	
3–4	9 (4.43)	3 (4.55)	3 (5.66)	3 (3.57)	
≥5	3 (1.48)	0 (0.00)	0 (0.00)	3 (3.57)	
History of mental illness (*N* = 198)					
Yes	72 (36.36)	24 (36.92)	19 (36.54)	29 (35.80)	χ^2^(2) = 0.021, *p* = 0.99
No	126 (63.64)	41 (63.08)	33 (63.46)	52 (64.20)	

**Table 2:** Means, Standard Deviations, and Group Comparisons for the Total Sample and the Subsamples of Pregnant Women (PW), Postpartum Women (PPW), and Non-Perinatal Female Controls (NPW).

Variable		Total Sample *N* = 203	PW *n* = 66	PPW *n* = 53	NPW *n* = 84	Statistics (ANOVA)
OCI-R	Total	15.438 (9.559)	14.773 (9.834)	15.925 (10.903)	15.655 (8.463)	*F* (2, 200) = 0.248, *p* = 0.780, ƞ_p_^2^ = 0.002
	Washing	2.207 (2.229)	2.227 (2.372)	2.434 (2.275)	2.048 (2.094)	*F* (2, 200) = 0.490, *p* = 0.613, ƞ_p_^2^ = 0.005
	Obsessing	3.360 (2.912)	3.000 (2.712)	3.170 (2.765)	3.762 (3.130)	*F* (2, 200) = 1.424, *p* = 0.243, ƞ_p_^2^ = 0.014
	Hoarding	2.803 (2.468)	2.742 (2.747)	3.094 (2.475)	2.667 (2.235)	*F* (2, 200) = 0.515, *p* = 0.598, ƞ_p_^2^ = 0.005
	Ordering	3.241 (2.909)	3.197 (2.824)	3.434 (3.237)	3.155 (2.783)	*F* (2, 200) = 0.160, *p* = 0.852, ƞ_p_^2^ = 0.002
	Checking	2.640 (2.241)	2.470 (2.573)	2.623 (2.132)	2.786 (2.036)	*F* (2, 200) = 0.367, *p* = 0.693, ƞ_p_^2^ = 0.004
	Neutralizing	1.187 (1.590)	1.136 (1.548)	1.170 (1.614)	1.238 (1.625)	*F* (2, 200) = 0.079, *p* = 0.924, ƞ_p_^2^ = 0.001
PHQ-9 ^a^		7.543 (5.183)	7.697 (4.781)	6.846 (4.526)	7.864 (5.866)	*F* (2, 196) = 0.652, *p* = 0.522, ƞ_p_^2^ = 0.007
GAD-7 ^b^		6.654 (4.540)	6.227 (4.790)	6.547 (4.074)	7.060 (4.636)	*F* (2, 199) = 0.636, *p* = 0.530, ƞ_p_^2^ = 0.006

*Note.* OCI−R = Obsessive–Compulsive Inventory—Revised; GAD-7 = Generalized Anxiety Disorder Screener-7; PHQ-9 = Patient Health Questionnaire-9. ^a^ PHQ-9: *N* = 199, with PW: *n* = 66, PPW: *n* = 52, and NPW: *n* = 81; ^b^ GAD-7: *N* = 202, with PW: *n* = 66, PPW: *n* = 53, and NPW: *n* = 83.

## Data Availability

The raw data supporting the conclusions of this article will be made available by the authors on request.

## Supplementary Materials

The following supporting information can be downloaded at: https://www.mdpi.com/article/10.3390/jcm15218665/s1. Table S1: Adjusted Group Comparisons of Obsessive-Compulsive, Anxiety, and Depressive Symptom Scores; Table S2: Pairwise Differences in the Proportions of Participants Meeting Exploratory OCI-R Score Thresholds; Table S3: Exploratory Subgroup Analyses of Obsessive-Compulsive, Anxiety, and Depressive Symptom Scores.
