# Supplementary material for: Obsessive-Compulsive Symptoms During Pregnancy and the Postpartum Period: A Cross-Sectional Comparison with Non-Perinatal Female Controls During the COVID-19 Pandemic
Source: J Clin Med. 2026 Nov 6;15(21):8665. doi: 10.3390/jcm15218665 (PMC13763624; doi:10.3390/jcm15218665)
Supplement: Supplementary file 1 [file jcm-15-08665-s001.zip › jcm-4608908-supplementary.pdf]

Table S1

Adjusted Group Comparisons of Obsessive-Compulsive, Anxiety, and Depressive Symptom Scores

Outcome	Model	N	F	df	p	Partial η^2
OCI-R total	1	203	0.316	2, 198	.730	.003
GAD-7	1	202	0.531	2, 197	.589	.005
PHQ-9	1	199	0.343	2, 194	.710	.004
OCI-R total	2	203	0.521	2, 197	.595	.005
GAD-7	2	202	0.556	2, 196	.575	.006
PHQ-9	2	199	0.179	2, 193	.836	.002

Note. Model 1 adjusted for age and relationship status. Model 2 additionally adjusted for number of children. The F statistics refer to the omnibus effect of group membership (pregnant women, postpartum women, and the non-perinatal female controls). All analyses were exploratory. OCI-R = Obsessive-Compulsive Inventory–Revised; GAD-7 = Generalized Anxiety Disorder–7; PHQ-9 = Patient Health Questionnaire–9; η^2 = eta squared.

Table S2

Pairwise Differences in the Proportions of Participants Meeting Exploratory OCI-R Score Thresholds

OCI-R threshold	Comparison	Group 1, n/N (%)	Group 2, n/N (%)	Difference (95% CI), pp
≥18	PW − PPW	22/66 (33.3)	18/53 (34.0)	-0.6 [-17.5, +15.8]
≥18	PW − NPW	22/66 (33.3)	32/84 (38.1)	-4.8 [-19.5, +10.6]
≥18	PPW − NPW	18/53 (34.0)	32/84 (38.1)	-4.1 [-19.7, +12.4]
≥28	PW − PPW	7/66 (10.6)	12/53 (22.6)	-12.0 [-26.0, +1.3]
≥28	PW − NPW	7/66 (10.6)	8/84 (9.5)	+1.1 [-8.7, +11.8]
≥28	PPW − NPW	12/53 (22.6)	8/84 (9.5)	+13.1 [+0.8, +26.8]

Note. PW = pregnant women; PPW = postpartum women; NPW = non-perinatal female controls; OCI-R = Obsessive-Compulsive Inventory–Revised; pp = percentage points; CI = confidence interval. Differences are calculated as the proportion in Group 1 minus the proportion in Group 2. Unadjusted 95% CIs for independent proportions were calculated using the Newcombe method based on Wilson score intervals. The comparisons are exploratory and are not adjusted for multiple testing. The OCI-R recall period was modified to seven days; therefore, thresholds of ≥18 and ≥28 are exploratory score categories rather than validated clinical cutoffs.

Table S3

Exploratory Subgroup Analyses of Obsessive-Compulsive, Anxiety, and Depressive Symptom Scores

Subgroup	Outcome	N	F	df	p
**Married or stable relationship**	OCI-R total	179	0.325	2, 176	.723
	Washing	179	1.292	2, 176	.277
	Obsessing	179	0.107	2, 176	.898
	Hoarding	179	0.584	2, 176	.559
	Ordering	179	0.282	2, 176	.755
	Checking	179	0.175	2, 176	.840
	Neutralizing	179	0.014	2, 176	.986
	GAD-7	178	0.165	2, 175	.848
	PHQ-9	175	0.812	2, 172	.446
**Monthly income > €1,000**	OCI-R total	172	0.327	2, 169	.721
	Washing	172	1.077	2, 169	.343
	Obsessing	172	0.159	2, 169	.853
	Hoarding	172	0.673	2, 169	.512
	Ordering	172	0.494	2, 169	.611
	Checking	172	0.481	2, 169	.619
	Neutralizing	172	0.111	2, 169	.895
	GAD-7	171	0.318	2, 168	.728
	PHQ-9	168	0.705	2, 165	.495
**Non-students**	OCI-R total	178	0.065	2, 175	.937
	Washing	178	1.394	2, 175	.251
	Obsessing	178	0.877	2, 175	.418
	Hoarding	178	0.256	2, 175	.774
	Ordering	178	0.217	2, 175	.805
	Checking	178	0.198	2, 175	.820
	Neutralizing	178	0.253	2, 175	.777
	GAD-7	177	0.237	2, 174	.789
	PHQ-9	174	0.607	2, 171	.546

Note. One-way ANOVAs compared pregnant women, postpartum women, and non-perinatal female controls within each restricted subsample. N varies by outcome because of missing data. df_2 denotes the within-group (error) degrees of freedom. All analyses were exploratory; p values are unadjusted. OCI-R = Obsessive-Compulsive Inventory–Revised; GAD-7 = Generalized Anxiety Disorder–7; PHQ-9 = Patient Health Questionnaire–9.
